# Exploring the potential of tobacco microorganisms for enhancing the flavor of naturally fermented essential oils

**DOI:** 10.3389/fmicb.2026.1780070

**Published:** 2026-10-01

**Authors:** Yan Jin, He Aiming, Hu Qi, Ye Zhuming, Zhang Yihan, Kan Qixin

**Affiliations:** 1 Hebei China Tobacco Industry Co., Ltd., Shijiazhuang, China; 2 Guangdong Provincial Key Laboratory of Nutraceuticals and Functional Foods, College of Food Sciences, South China Agricultural University, Guangzhou, Guangdong, China

**Keywords:** aroma-producing microorganisms, fermentation characteristics, metabolic pathways, sensory analysis, volatile organic compounds

## Abstract

To isolate and characterize aroma-producing microorganisms from tobacco and evaluate their fermentation potential, 12 strains obtained from tobacco leaves and root soil were identified as members of the genera Enterobacter, Burkholderia, and Pseudomonas. High amylase activity was detected in most Enterobacter and Pseudomonas strains, whereas low cellulase activity and limited ethanol tolerance were generally recorded across all isolates. A total of 174 volatile compounds were detected by HS-SPME-GC–MS, among which alcohols were the most abundant class. Seventy-four odor-active compounds were identified by relative odor activity value analysis, with ketones (2,3-butanedione) and aldehydes ((E, E)-2,4-Decadienal, (E)-2-nonenal) being highlighted as key contributors to sweetness, fatty, and green aroma. The strains were categorized into two metabolic aroma types: ketone type, aldehyde type, which were associated with pyruvate and lipoxygenase pathways. It was demonstrated that microbial metabolic specificity determines aroma profiles, thereby providing a foundation for targeted flavor design through strategic strain selection.

## 1 Introduction

Microbial ecology is now an integral component of modern agricultural ecosystems, directly influencing the quality of agricultural products (Liu et al., 2021). Among the sensory profile of agricultural products, flavor is a critical determinant of consumer acceptance and commercial value (Berger, 2009). The formation of this complex flavor is influenced not only by plant genetics and nutritional conditions, but also significantly by dynamic interactions with diverse associated microbial communities (Jiang et al., 2025; Raina et al., 2025; Yan et al., 2020). Accumulating evidence has established that plant-associated microbiomes are recognized as key contributors to the biochemical pathways involved in flavor compound synthesis in various crops, including rice, grapes, roses, raspberries, coffee, tea, and truffles (Chen et al., 2023; Mocali et al., 2020; Pregolini et al., 2021; Raina et al., 2025; Sangiorgio et al., 2022; Shi et al., 2024). Commensal microorganisms utilize their vast metabolic repertoire to catalyze the breakdown of complex plant compounds into precursors, which are subsequently transformed into a wide spectrum of volatile organic compounds. As shown in examples, symbiotic rhizobia can increase the content of aromatic compounds 2-acetyl-1-pyrrolin in Thai jasmine rice during the growth process (Chinachanta et al., 2021). In grape growing areas, the increase of soil actinomycetes content promotes the enrichment of sulfur and oxygen in the soil, and the activity of arylsulfatase is detected at a higher level, so as to regulate the physiological characteristics of grape plants and improve the flavor and quality of grapes (Mocali et al., 2020). Nowadays, the mining of new and stable microorganisms from specific agricultural ecosystems has become a frontier direction in biotechnology.

Tobacco (*Nicotiana tabacum* L.), a crop of significant economic and cultural importance, is considered a highly relevant model system for investigating plant–microbe–aroma interactions (Hu et al., 2025). Tobacco flavor quality is an exceptionally complex trait, which is determined not only by the plant’s genetic background and post-harvest curing processes, but also by its dynamic interactions with associated microbial communities (Hao et al., 2025). A diverse community of bacteria and fungi is hosted by tobacco leaves and roots. This microbial community forms a metabolic reservoir capable of synthesizing a wide spectrum of volatile organic compounds, which significantly contribute to the characteristic aroma profile of the plant (Yan et al., 2025). It has been reported that the microbial abundance in fresh tobacco leaves ranges from 2 × 103 to 7 × 105 CFU/g, while the number of microorganisms present can reach up to 8 × 105 CFU/g. More notably, a marked divergence has been observed between the microbiota of flue-cured and cigar tobacco: the former is predominantly dominated by Bacillus and Pseudomonas, whereas the latter is primarily colonized by Lysobacter and Brevibacterium (Hu et al., 2025). Importantly, the inoculation of tobacco leaves with specific bacteria, such as Bacillus species, has been demonstrated to increase the concentrations of characteristic aroma compounds, including 2-methoxy-4-vinylphenol and 2-methylhexanoic acid (Shan et al., 2025). These results confirm the role of these microbes as key agents driving aroma formation underscoring how distinct microbial profiles lead to differences in secondary metabolites and ultimately shape the final flavor profile.

In recent years, a growing industrial demand has been observed across the daily chemical sectors for microbial starters that can efficiently produce natural flavor compounds through fermentation processes (Berger, 2009). Traditional processes often rely on a limited set of standard strains, such as *Saccharomyces cerevisiae*, *Escherichia coli*, which may not fully exploit the diverse aromatic potential of raw substrates (Budner et al., 2024; Wang et al., 2025). Therefore, the bioprospecting of novel, non-conventional microorganisms from unique ecological niches has become a pivotal strategy within biotechnology for developing innovative fermentation processes that yield enhanced or new flavor profiles. Microbes associated with aromatic plants such as tobacco to metabolize complex substrates and equipped with unique enzymatic arsenals, present ideal candidates for such endeavors (Ning et al., 2023; Raina et al., 2025).

The systematic isolation and functional screening of tobacco-derived microbes based on key fermentation traits have not been thoroughly investigated. Similarly, a direct and objective evaluation of their aroma-forming potential in non-tobacco food matrices has also received limited attention. To bridge this gap, this study aimed to isolate, screen, and identify bacteria and fungi with high aroma-producing potential from the tobacco leave and root in Nanxiong City, Guangdong Province, China. The isolates were enriched and cultured using selective media, followed by molecular identification via ITS/16S rRNA sequencing. Key fermentative characteristics were evaluated, including amylase, cellulase activities and ethanol tolerance. Promising strains were subsequently applied as starter cultures in a corn-based fermentation model to empirically assess their influence on the aroma profile. The fermented products underwent sensory evaluation and headspace solid-phase microextraction coupled with gas chromatography–mass spectrometry (HS-SPME-GC–MS). The resulting volatile organic compounds data were further analyzed using relative odor activity value (ROAV) to scientifically determine the contribution of individual compounds to the overall aroma. Additionally, the potential aroma metabolism mechanisms of these strains were inferred.

## 2 Materials and methods

### 2.1 Materials

As a recognized geographical specialty of Guangdong Province, Nanxiong sun-cured tobacco is characterized by its golden leaf color and a mellow, aromatic flavor profile. Tobacco plant and soil samples were collected from Hengfen New Village, Shuikou Town, Nanxiong City, Shaoguan, Guangdong Province (Longitude 114.49°E, Latitude 25.15°N). Yeast Extract Peptone Dextrose Medium (YPD), Mueller-Hinton Medium (MH), Potato Dextrose Agar (PDA), starch agar medium, and Congo red cellulose medium were purchased from Shanghai Yingwei Jieji Biotechnology Co., Ltd. Lugol’s iodine solution, physiological saline, ethanol, and 2-octanol were obtained from Shanghai Macklin Biochemical Co., Ltd. Fresh corn kernels were acquired from a local market.

### 2.2 Collection of tobacco leaves and roots

To ensure sample representativeness, collection sites were selected based on established sampling protocols (Hu et al., 2025). Areas potentially influenced by edge effects, such as field margins, roadsides, and other transitional zones, were avoided. Soil samples were taken from locations showing vigorous plant growth and low weed density. A five-point sampling method was employed in each sampling area. Surface debris was cleared before soil collection. Topsoil samples (0–20 cm depth) were gathered from around the base of healthy tobacco plants using a sterile soil corer. Samples from each point were mixed to form a composite sample per site. Fully expanded leaves were aseptically collected from healthy, symptom-free plants at a uniform growth stage. All samples were promptly placed in sterile bags, labeled, and stored on ice for transport to the laboratory.

### 2.3 Strain isolation and cultivation

Referring to the sampling protocol (Hu et al., 2025), the following procedures were performed as shown in Figure 1A: ① Strain Isolation: Approximately 5 g of tobacco leaf sample was cut into small pieces and transferred to a sterile beaker containing 50 mL of physiological saline. The mixture was shaken at 150 rpm for 30 min and then filtered through sterile filter paper. The filtrate was equally divided into three portions, each inoculated into 100 mL of one of the following liquid media: YPD, MH, or PDA, in sterile conical flasks. The same procedure was applied to soil samples: 5 g of crushed soil was suspended in saline, shaken, filtered, and the filtrate was used to inoculate the three media. All flasks were incubated at 30 °C for 18–24 h with shaking at 180 rpm. ② Streak Plate Isolation: After enrichment, a loopful of culture was taken from each flask and streaked onto the corresponding solid medium (YPD, MH, or PDA agar) using a sterile inoculation loop. The plates were incubated at 30 °C until well-isolated single colonies appeared. ③ Single Colony Picking: Distinct single colonies were selected and inoculated into fresh liquid medium of the same type. The cultures were incubated at 30 °C with shaking until the optical density at 600 nm (OD600) reached approximately 0.5. These cultures were used as seed inocula for subsequent fermentation experiments. ④ Glycerol Storage: For long-term preservation, 2 mL of seed culture was mixed with an equal volume of 50% sterile glycerol in a cryovial. The vials were stored at −80 °C.

**Figure 1:**
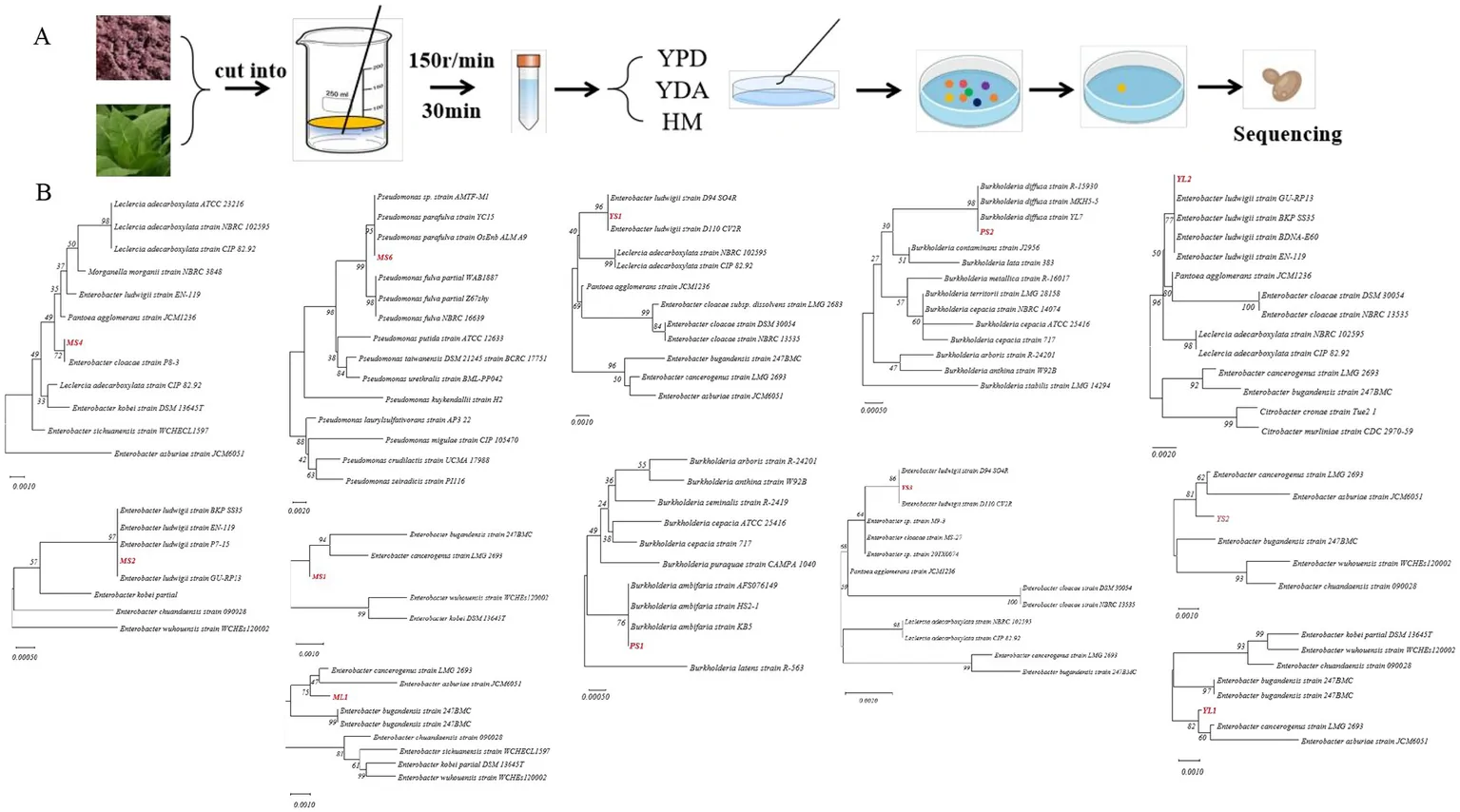
Microbial isolation and identification of tobacco samples. **(A)** Flow chart of experimental sampling and analysis. **(B)** Phylogenetic tree of tobacco source strains.

### 2.4 Sequencing of ITS and 16S rRNA from isolated strains

Fungal DNA was extracted using the cetyltrimethylammonium bromide (CTAB) method with slight modifications based on a previously described protocol (Shi et al., 2024). Approximately 100 mg of fungus was transferred to a 2 mL sterile microcentrifuge tube and homogenized with 800 μL of pre-warmed CTAB extraction buffer using a sterile micropestle. The homogenate was incubated at 65 °C for 30 min in a water bath. An equal volume of chloroform–isoamyl alcohol (24:1, v/v) was added, and the mixture was centrifuged at 12,000 × g for 10 min at 4 °C. The upper aqueous phase was carefully transferred to a new tube. Nucleic acids were precipitated by adding 0.7 volumes of isopropanol, followed by incubation at −20 °C for 1 h. The DNA pellet was washed twice with 1 mL of 70% (v/v) ethanol, air-dried, and resuspended in 50 μL of nuclease-free water.

Bacterial DNA was extracted using a modified enzymatic lysis protocol. Cell pellets from pure cultures were resuspended in 567 μL of Tris-EDTA buffer. Then, 30 μL of 10% (w/v) Sodium Dodecyl Sulfate (SDS) and 15 μL of proteinase K (20 mg/mL) were added, followed by incubation at 55 °C for 30 min. An equal volume of chloroform–isoamyl alcohol (24:1) was added, and the mixture was gently inverted for 2 min before being centrifuged at 12,000 × g for 5 min. The supernatant was transferred to a new tube, and DNA was precipitated by adding an equal volume of isopropanol. The pellet was washed with 70% ethanol, air-dried, and finally dissolved in 50 μL of TE buffer or nuclease-free water.

PCR amplification was performed using TransStart^®^ FastPfu DNA Polymerase (TransGen Biotech) under stringent conditions to ensure specificity and reproducibility. The 50 μL reaction mixture contained the components specified in Supplementary Table S1. Amplification was carried out in an ABI GeneAmp^®^ 9,700 thermal cycler (Applied Biosystems). The cycling protocol for the bacterial 16S rRNA gene consisted of an initial denaturation at 95 °C for 5 min; followed by 27 cycles of denaturation at 95 °C for 30 s, annealing at 55 °C for 30 s, and extension at 72 °C for 45 s; with a final extension at 72 °C for 10 min. For the fungal ITS region, 29 cycles were used under identical thermal conditions. All PCR products were stored at 10 °C until further analysis by agarose gel electrophoresis and purification.

### 2.5 Determination of fermentation characteristics of isolated strains

#### 2.5.1 Gram staining of bacterial strains

In this study, the bacterial smear was prepared and fixed by heat. Subsequently, the sample was stained with crystal violet for 1 min, followed by iodine solution for 1 min. It was then decolorized with 95% alcohol for approximately 30 s, or until the runoff became colorless, and counterstained with safranin for 1 min. After rinsing with water and air-drying, the slide was examined under an optical microscope. Images of the stained bacteria were captured at a magnification of 100 times.

#### 2.5.2 Amylase and cellulase activities

When the strains reached the logarithmic growth phase, the bacterial solution was diluted with fresh culture medium to adjust the OD600 to 0.1. Then, 10 μL of the bacterial suspension was spotted onto the surface of solid starch hydrolysis agar and cellulose Congo red agar plates. Under aseptic conditions, the plates were air-dried and incubated at 30 °C for 72 h. For amylase activity, 1–2 mL of 5% Lugol’s iodine solution was evenly applied to the plate surface for staining. After 5 min, the plate was rinsed with physiological saline, and the diameter of the transparent zone was accurately measured using the cross method. For cellulase activity, the diameter of the transparent circle was directly measured by the same cross method to evaluate enzymatic activity.

#### 2.5.3 Determination of ethanol tolerance

Ethanol tolerance of the isolated strains was evaluated using a liquid medium assay. First, the strains were inoculated into ethanol-free liquid medium and cultured until the logarithmic growth phase was reached. Then, ethanol was added to final concentrations of 8, 10, and 12%. Samples were collected at various time intervals, and the optical density (OD) of the culture was measured. The growth and ethanol tolerance of the strains under different ethanol concentrations were assessed by comparing the OD values over time.

### 2.6 Sensory evaluation of fermentation experiments

In the sweet corn fermentation experiment, fresh sweet corn kernels were crushed, and 20 g of the crushed material was weighed into bottles. The bottles were placed in an autoclave and steamed at 121 °C for 30 min. After cooling, 5 mL of sterile water and 0.4 mL of yeast seed culture were added to each sample. Three control groups were set up, each receiving 5 mL of sterile water and 0.4 mL of liquid medium instead of the yeast seed culture. All samples were fermented at a constant temperature of 30 °C, and sensory evaluations of odor were conducted every 24 h until 7 day. The sensory evaluation process involved five experienced panelists to ensure reliability and consistency of the results. To minimize external interference, samples were placed in transparent, colorless containers to eliminate potential effects of the container on odor perception. The panelists opened the sample containers and gently shook them to promote uniform release of volatile compounds. Each evaluation lasted 2–3 min to ensure accuracy and prevent olfactory fatigue. Odor intensity was rated on a nine-point scale, with scores assigned based on the perceived concentration of the odor. Appropriate agreements were established to protect the rights and privacy of all participants. The materials used in the study consisted of safe, ordinary food items that complied with national standards. In accordance with institutional and national regulations, ethical approval was not required for this human sensory study, as such research is exempt from ethical review under current policies. Written informed consent was obtained from all participants prior to the study, and all procedures strictly adhered to regulations protecting participants’ rights and interests.

### 2.7 Characterization of aroma compounds by GC–MS

In order to analyze the volatile aromatic compounds produced during the fermentation of sweet corn, this study referred to the scheme of literature and modified it appropriately (Zhu et al., 2025). 6 g of fermented sample was weighed, and 0.5 g of sodium chloride along with 1 μL of internal standard solution (2-octanol, 0.001 mg/mL) were added and thoroughly mixed. The prepared sample was equilibrated at 80 °C for 10 min in a constant-temperature incubator. A CAR/PDMS/DVB SPME fiber was then inserted for headspace extraction over 50 min. After extraction, the fiber was introduced into the GC–MS injection port and desorbed at 250 °C for 5 min. Chromatographic separation was performed using a DB-WAX capillary column (60 m × 250 μm × 0.25 μm) with high-purity helium as the carrier gas at a constant flow rate of 1 mL·min-1. The inlet was operated in splitless mode at a pressure of 112 kPa. The temperature program was set as follows: initial temperature of 40 °C held for 5 min, increased to 120 °C at 3 °C·min-1 and held for 1 min, then raised to 180 °C at 5 °C·min-1 and held for 1 min, and finally elevated to 230 °C at 10 °C·min-1 and held for 5 min. The mass spectrometry conditions were as follows: electron impact (EI) ion source with an electron energy of 70 eV; ion source temperature of 230 °C; interface temperature of 210 °C; quadrupole temperature of 150 °C; and a mass scan range of 33–500 m/z.

### 2.8 Analysis of volatile compounds by relative odor activity value

The relative Odor Activity Value (ROAV) is a recently introduced parameter used to assess the contribution of volatile compounds to flavor (CUI et al., 2010; Xie et al., 2023). This method integrates odor thresholds to identify key aroma compounds, thereby clarifying the role of individual substances in the overall aroma profile of a sample. Compounds with ROAV values equal to or more than 1 are classified as key aroma components. Volatile compounds with ROAV ≤ 1 are generally considered to have minimal influence on the overall flavor (Fang et al., 2024). The formulas used for calculation are as follows:


OAV=Ci/OTi

ROAV=100×OAVi/OAVmax

Odor threshold (OT) values for the volatile compounds in water were obtained from previously published literature and reference books.

### 2.9 Data processing and analysis

All experiments were performed in triplicate. Data were statistically analyzed using SPSS 24.0 software. Significance was determined based on Duncan’s multiple range test, with a significance level set at *p* < 0.05 indicating a significant difference and *p* < 0.01 indicating an extremely significant difference. Results are expressed as mean ± standard deviation. Volatile compounds identification was performed using retention index matching (C_7_–C_30_, n-alkanes), mass spectral matching against the NIST 20 library (similarity ≥80%, with manual verification of key spectra). Multivariate statistical analysis, along with KEGG enrichment and pathway analysis, was conducted using MetaboAnalyst 6.0.

## 3 Results and discussion

### 3.1 Isolation and identification of aroma-producing microorganisms

ITS and 16S rRNA sequencing are used for microorganism identification as methods which can generally accurately identify target fungi or bacteria to the genus level (Shan et al., 2025; Shi et al., 2024). Twelve aroma-producing strains were isolated from tobacco leaves and roots, which were identified through ITS and 16S rRNA gene sequencing in Table 1. The result of phylogenetic tree showed that they belonged to the genera Enterobacter, Burkholderia, and Pseudomonas, as detailed in Figure 1B. Among them, strains ML1 and YL1, isolated from leaves, were identified as members of the genus Enterobacter, and YL2 was classified as *Enterobacter ludwigii*. The genus Enterobacter is a type of facultatively anaerobic and gram-negative bacilli that are widely distributed in diverse environments such as plants, water, and animals. Members of this genus are characterized by facultative anaerobic metabolism and sugar fermentation capability (Chinachanta et al., 2021). During tobacco leaf aging, Enterobacter accounts for 13.60–39.82% of the total bacterial population, and the content of neutral flavor components such as ketones and aldehydes significantly increases in the aged tobacco leaves (Huang et al., 2022). Among the strains isolated from soil, nine species were identified, with the genus Enterobacter (6 species) being the most predominant. The Enterobacter strains were classified as follows: MS4 (*Enterobacter cloacae*), MS1 (Enterobacter), MS2 (*Enterobacter ludwigii*), YS1 (*Enterobacter ludwigii*), YS2 (Enterobacter), and YS3 (*Enterobacter ludwigii*). *Enterobacter ludwigii* have been proven by numerous studies to be an effective plant growth promoter, which can effectively break down the starch component in tobacco (Hu et al., 2024). In addition, *Enterobacter ludwigii* can enhance the ability of plants to accumulate biomass, regulate osmotic pressure, improve photosynthetic efficiency, reduce damage caused by salt stress, and change the structure of root microbial community (Wei et al., 2022). The genus Burkholderia was represented by two species: PS1 (*Burkholderia ambifaria*) and PS2 (*Burkholderia diffusa*). Both *Burkholderia ambifaria* and *Burkholderia diffusa* are characterized by high metabolic diversity, enabling the utilization of a wide range of carbon sources. They are commonly found in plant rhizosphere soils (Timofeeva et al., 2023). Both species are recognized for their functional roles, including nitrogen fixation, phosphorus solubilization, and biocontrol. The aroma-producing characteristics of these strains are attributed to their active metabolism, often associated with the production of sulfur-containing compounds and fruity ester aromas (Timofeeva et al., 2023). The genus Pseudomonas is represented by one species: MS6 (*Pseudomonas parafulva*). *Pseudomonas parafulva* is characterized as an aerobic, non-fluorescent Pseudomonas species with complex metabolic capabilities. A notable metabolic feature is its efficient degradation of a wide range of complex organic compounds include nicotine. The aroma-producing characteristics of this species are primarily characterized by pungent odors resembling sulfur, onion, or garlic (Hu et al., 2025; Yu et al., 2025).

**Table 1:** Identification and fermentation characteristics of aroma-producing microorganisms.

Number	Origin	Genus	Species^A^	Cellulase activities^B^	Amylase activities^C^	Ethanol concentrations^D^			
						0%	8%	10%	12%
MS4	Root	*Enterobacter*	*cloacae*	−	1.2	+	−	−	−
MS6	Root	*Pseudomonas*	*Parafulva*	−	0.9	+	−	−	−
ML1	Leave	*Enterobacter*	*−*	−	1.5	+	−	−	−
MS1	Root	*Enterobacter*	*−*	−	−	+	−	−	−
MS2	Root	*Enterobacter*	*ludwigii*	−	−	+	−	−	−
PS1	Root	*Burkholderia*	*ambifaria*	−	0.8	+	−	−	−
PS2	Root	*Burkholderia*	*diffusa*	−	0.8	+	−	−	−
YL1	Leave	*Enterobacter*	*−*	−	−	+	−	−	−
YS1	Root	*Enterobacter*	*ludwigii*	−	−	+	−	−	−
YL2	Leave	*Enterobacter*	*ludwigii*	−	−	+	−	−	−
YS2	Root	*Enterobacter*	*−*	−	1.2	+	−	−	−
YS3	Root	*Enterobacter*	*ludwigii*	−	1.0	+	−	−	−

^A^indicated the absence of bacterial species identification results; ^B^indicated the absence of cellulase activities in the bacterial species; ^C^Data represents the size of the amylase activity zone; ^D^+ indicated the bacterial can survive; − indicated the bacterial did not survive.

### 3.2 Study on fermentation characteristics of aroma-producing microorganisms

In this study, the Gram staining method was applied to each isolated strain, and the cellular morphology was observed under an optical microscope. As shown in Figure 2A, all strains exhibited Gram-negative characteristics in red, consistent with the species identification results. YL1, ML1, MS1, and YS2 belonged to the genus Enterobacter, typically occurring singly or in pairs. Their short rod-shaped cells stained red and appeared either dispersed or slightly clustered. YS1, MS2, YL2, and YS3 were identified as *Enterobacter ludwigii* and displayed morphology similar to that of other Enterobacter species. MS4, also classified as *Enterobacter cloacae*, presented a straight rod-shaped morphology. PS1 and PS2 correspond to *Burkholderia ambifaria* and *Burkholderia diffusa*, respectively, both of which are short rods. MS6 was identified as *Pseudomonas parafulva*, characterized by slender, polar-flagellated rod-shaped cells, occurring either individually or in clusters.

**Figure 2:**
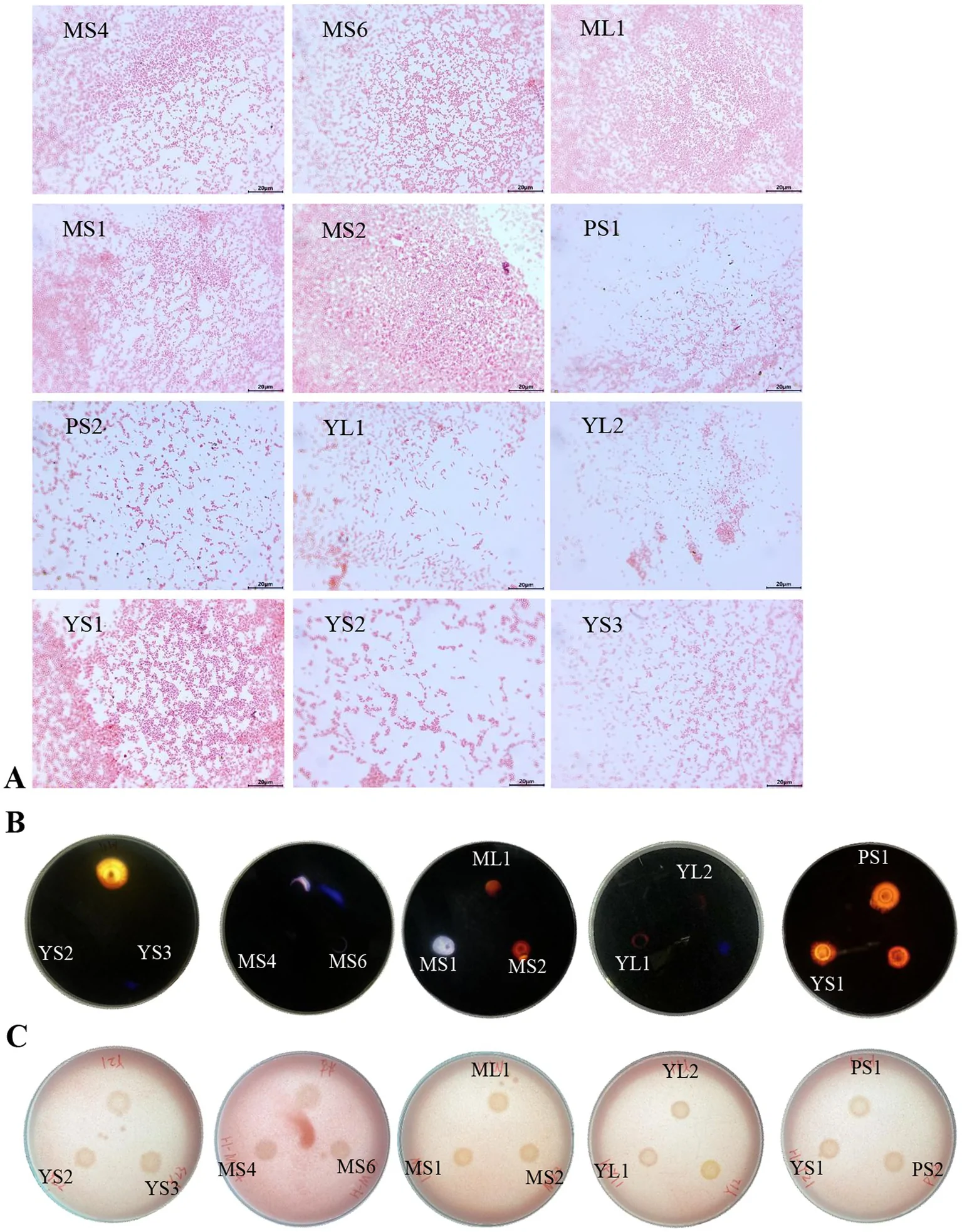
Analysis of microbial fermentation characteristics. **(A)** Microscopic staining. **(B)** amylase activity. **(C)** cellulase activity.

In the study of fermentation characteristics of aroma-producing strains, amylase activity, cellulase activity, and ethanol tolerance are considered three key indicators that collectively determine the industrial application potential of the strains (Behera et al., 2018). Amylase activity reflects the ability of a strain to degrade starch into fermentable sugars, which is essential for providing substrates for fermentation and initiating sugar metabolism (Lai et al., 2022). As shown in Table 1, relatively high amylase activity was observed in strains ML1, MS4, YS2, and YS3, whereas MS6, PS1, and PS2 demonstrated relatively low activity. No amylase activity was detected in strains MS1, MS2, YL1, YS1, or YL2 in Figure 2B. Cellulase activity is indicative of the capability of strains to utilize plant fibrous materials, enabling the range of raw material sources to be broadened, the release of flavor compounds to be enhanced, and the fermentation process to be improved (Lai et al., 2022). At present, cellulolytic activity is generally absent among the identified aroma-producing strains in Figure 2C. Ethanol tolerance is used to evaluate the survival and metabolic capacity of strains in an alcoholic environment (Lai et al., 2022). No growth was observed for the identified aroma-producing strains in ethanol concentrations exceeding 8%, which reflects their limited survival capacity and physiological stability under ethanol-induced stress. In high-concentration alcohol fermentation, ethanol tolerance is considered a critical screening criterion for strain selection. Only strains exhibiting high tolerance can ensure the smooth progression of the fermentation process and the stability of the final product’s characteristics (Lai et al., 2022). Amylase activity, cellulase activity, and ethanol tolerance are indirectly yet critically linked to aroma compound production and primarily determine the industrial application potential of microbial strains. Amylases and cellulases hydrolyze substrates (e.g., starch, cellulose, and hemicellulose) into fermentable sugars, thereby supplying the carbon skeletons necessary for the synthesis of aroma precursors, though these enzymes do not directly catalyze the formation of aromatic compounds. Ethanol tolerance enables strains to sustain essential metabolic activity during the mid-to-late stages of fermentation, thereby supporting the continued synthesis of esters and other aroma substances. Consequently, while these traits do not directly define the aromatic profile, they represent core engineering properties that ensure efficient, stable, and scalable aroma production, serving as key screening criteria for assessing the suitability of strains in industrial fermentation processes.

### 3.3 Sensory analysis of fermentation products of different bacterial species

A comprehensive analysis of fermentation products from 12 strains was conducted using the quantitative descriptive analysis method, evaluating seven sensory attributes: fruity, floral, mellow, ester-like, sour, sweet, and fresh. As shown in Figure 3A, significant differences in flavor profiles among the strains were observed. Among these attributes, ester-like and sweet aromas were identified as common dominant characteristics. The strains were categorized into two distinct flavor types. The first type, represented by YS1, MS6, YL1, was characterized by prominent mellow and ester-like aromas, resulting in a rich and robust flavor profile. The second type, represented by PS2, MS1, MS2, MS4, YS2, YL2, YS3, ML1, PS1, was distinguished by distinct floral and fresh aroma, yielding a refreshing and vibrant aromatic style. Overall, the strains screened in this study were demonstrated to exhibit diverse and distinctive flavor characteristics.

**Figure 3:**
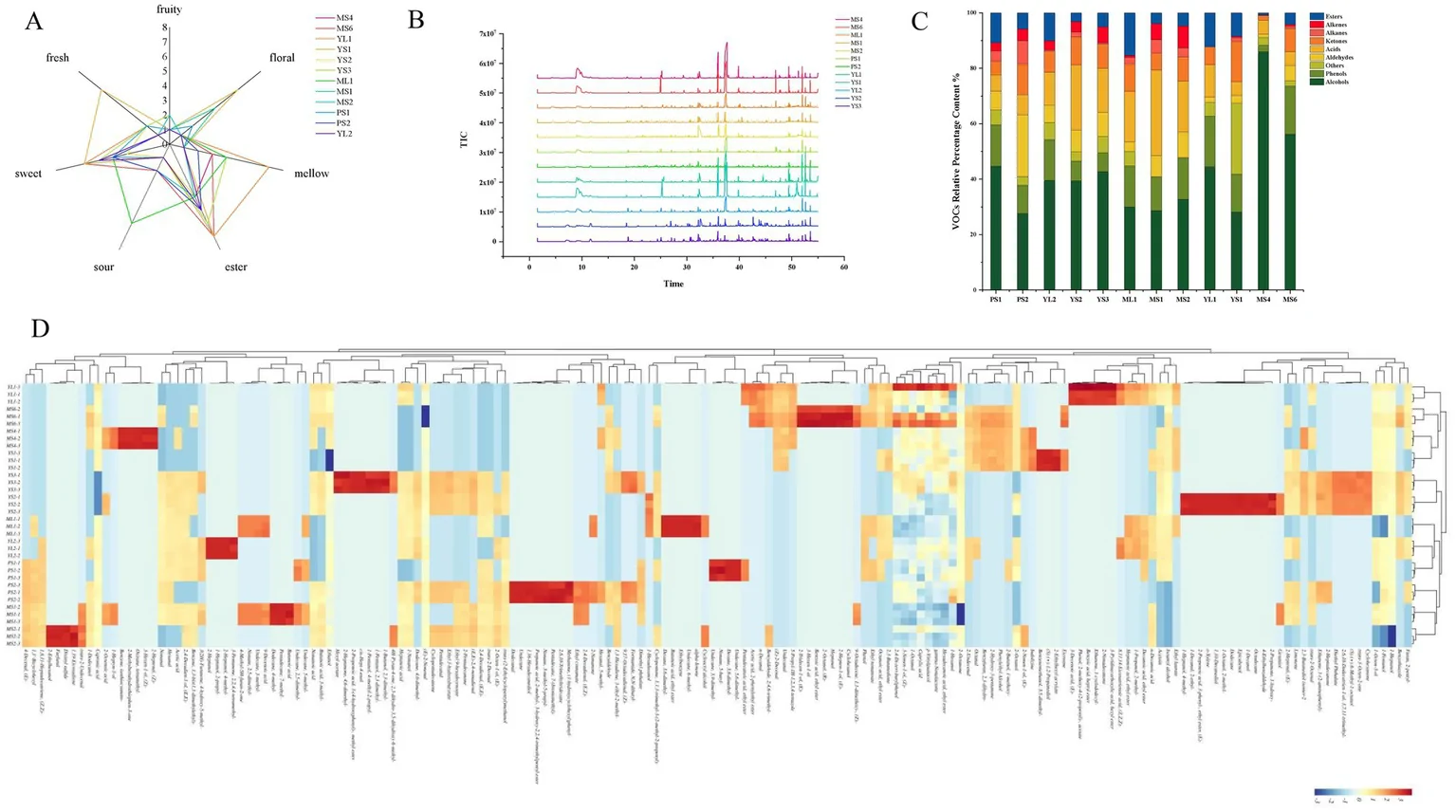
Volatile components in fermented products. **(A)** The radar chart of sensory evaluation. **(B)** Total ion chromatograms of fermentation products from different bacterial strains. **(C)** Total relative abundance. **(D)** Volatile organic compounds clustered heat map.

### 3.4 HS-SPME-GC–MS analysis of volatile components in fermented products of different bacterial species

#### 3.4.1 The analysis of volatile organic compounds

To comprehensively characterize the volatile organic compounds in the fermentation products of different strains, the volatile organic compounds were analyzed using headspace solid-phase microextraction coupled with gas chromatography–mass spectrometry (HS-SPME-GC–MS). The total ion current was shown in Figure 3B. A total of 174 volatile compounds were detected using GC–MS, spanning nine major categories: 48 alcohols, 26 aldehydes, 22 ketones, 22 alkanes, 22 esters, 12 acids, 7 alkenes, 4 phenols, and 11 other compounds. Detailed results are summarized in Supplementary Table S2.

The total volatile organic compound content was compared across different strains. Strains MS4 and MS6 were found to produce VOC contents of 70.85 μg/g and 69.96 μg/g, respectively, whereas the VOC yields from strains ML1, MS1, and MS2 ranged from 5.38 to 6.94 μg/g. It is noteworthy that alcohols were identified as the most abundant category, predominating significantly in quantity in Figure 3C. A total of 48 alcoholic compounds were detected across all samples. Representative compounds included 2,3-butanediol and ethanol. The highest concentration of 2,3-butanediol was detected in sample MS4 (40.1 μg/g), while notable accumulation of ethanol was observed in sample MS6 (26.02 μg/g). This prominent feature suggested that alcohols may represent the main products of carbohydrates fermentation or metabolic pathways, contributing substantially to the overall volatile profile (Yu et al., 2025). A total of 26 aldehydes were identified, including nonanal and (E)-2-nonenal. Nonanal was particularly abundant in sample MS6 (1.31 μg/g), while (E)-2-nonenal was widely distributed across multiple samples. This pattern indicated a potential origin from lipid oxidation and a possible role in imparting characteristic green or fatty aroma (Hao et al., 2025). In total, 22 ketones were detected, which were categorized into both aliphatic and heterocyclic types, which included 2,3-butanedione and 2(3H)-furanone, dihydro-5-pentyl-. Although ketones were not the most abundant compounds, they were found to contribute unique aromatic attributes. For instance, 2(3H)-furanone, dihydro-5-pentyl- was consistently detected in multiple samples, suggesting its importance in flavor complexity. A total of 22 esters were identified, primarily characterized as fatty acid esters. A relatively high concentration of ethyl hexadecanoate was measured in sample YL1 (5.06 μg/g), highlighting esterification as a key pathway in volatile compound formation (Hao et al., 2025). Acids, including acetic acid and octanoic acid, were also identified. Acetic acid reached a concentration of 1.85 μg/mL in sample YS2. These compounds may impart an acidic character to the samples and influence the overall sensory balance. Alkenes (7 species) and phenols (4 species) were present at low concentrations but were recognized as functionally important. Limonene and 2-methoxy-4-vinylphenol were associated with floral or smoky aroma (Wang et al., 2023; Zhu et al., 2025), with the latter being particularly high in sample YL1 (9.11 μg/g). Additionally, alkanes were predominantly identified as saturated hydrocarbons, such as undecane and dodecane. As shown in Figure 3D, all volatile organic compounds were visualized on a heat map to display their concentration variations. Through clustering analysis, the Fermentation samples can be broadly categorized into two groups: Group 1 (YL1, MS6, MS4, YS1); and Group 2(YS3, YS2, ML1, YL2, PS1, PS2, MS1, MS2). This distinct clustering pattern clearly reflected the underlying differences in metabolic profiles among the studied strains.

#### 3.4.2 Multivariate statistical analysis

The volatile compounds determined by GC–MS were analyzed using Partial Least Squares–Discriminant Analysis (PLS-DA). The parameters of the PLS-DA model (R^2^Y = 0.995, Q^2^ = 0.965) demonstrated strong explanatory and predictive capability. The score plot revealed distinct clustering and separation trends among different ferment sample groups within the principal component space. All samples were broadly divided into four groups: YS1, YL1, YL2, MS6, MS4, PS1 formed one group; PS2 formed another group; YS3, YS2 formed a third group; MS1, MS2, ML1 formed the fourth group. It indicated significant differences in volatile compound composition between groups along with good intra-group reproducibility in Figure 4A. Through combined analysis of VIP scores in Figure 4B, key volatile compounds responsible for inter-group differences were identified. In the VIP score plot, compounds with VIP values greater than 1.0 were found to contribute significantly to inter-group classification and were regarded as core markers distinguishing different sample groups (Xie et al., 2023). Key volatile compounds included 1,1’-Bicyclohexyl, 9-Decenoic acid, Capronic acid, Limonene, Furan, 2-pentyl- among others. These compounds were highly ranked in the VIP score plot (VIP > 2.0) and displayed distinct content variations across groups in the heatmap, which can be used to determine the source of fermentation strains.

**Figure 4:**
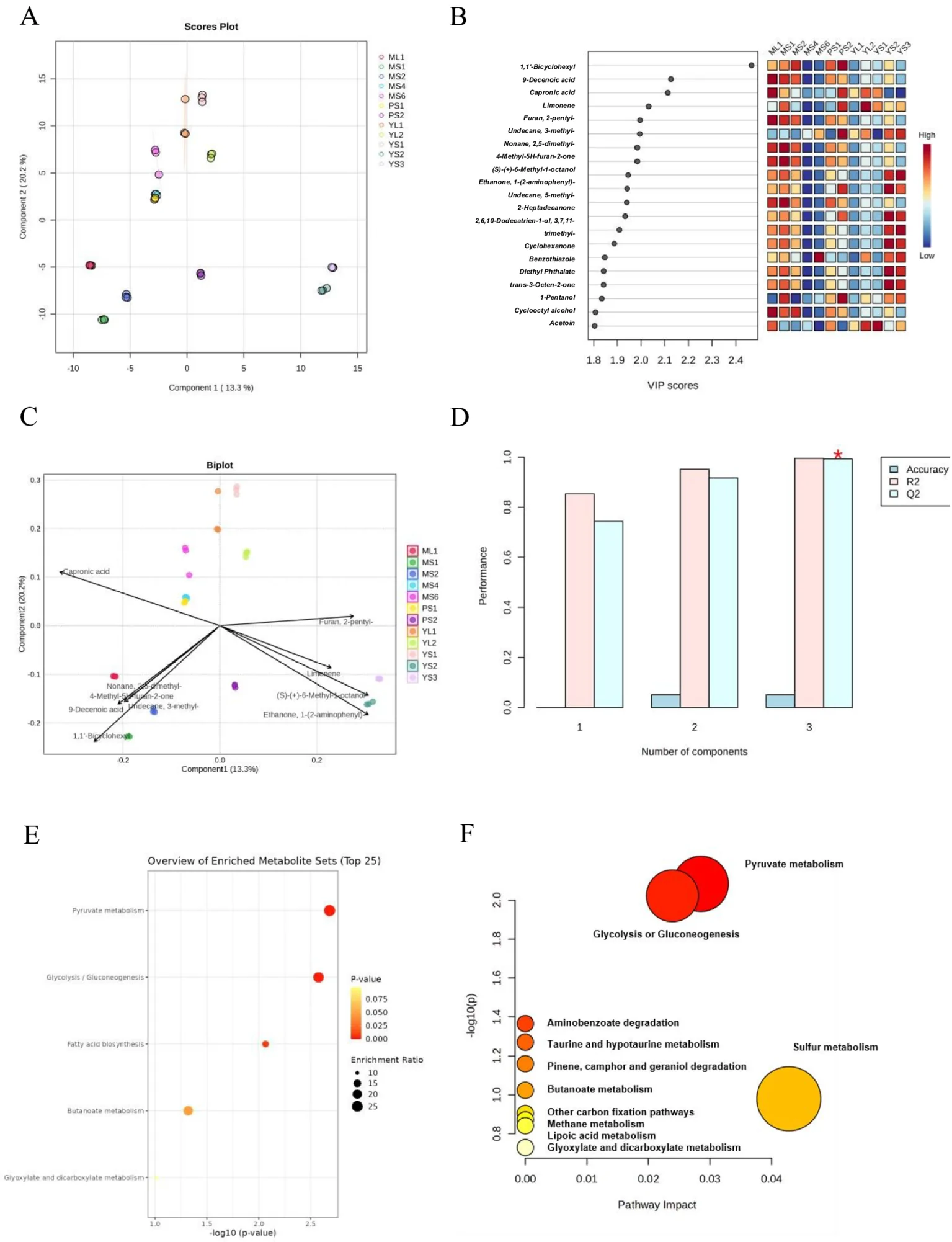
PLS-DA multivariate statistical analysis. **(A)** PLS-DA scores plot. **(B)** The VIP values of key VOCs. **(C)** Load diagram. **(D)** Cross validation score diagram. **(E)** The enrichment analysis of characteristic aroma component. **(F)** The pathway analysis of characteristic aroma component.

Furthermore, the direction of compound vectors was closely related to the spatial position of sample groups. The relationship between samples and compounds was illustrated through a biplot, which reflected both inter-group and intra-group differences (Shi et al., 2022). Such as capronic acid, Furan, 2-pentyl, Limonene, (S)-(+)-6-Methyl-1-octanol, Ethanone, 1-(2-aminophenyl), 1,1’-Bicyclohexyl, 9-Decenoic acid, Undecane, 3-methyl-, 4-Methyl-5H-furan-2-one, Nonane, 2,5-dimethyl-, they were found to contribute more significantly to principal component variation and identified as core volatile compounds driving sample group separation in Figure 4C. In the cross-validation results of partial least squares-discriminant analysis (PLS-DA), an increase in the number of components significantly improved the model’s fitting and predictive performance in Figure 4D. R^2^ measures the model’s ability to explain variance in the data. A value closer to 1 indicated better goodness-of-fit (Saccenti and Timmerman, 2016). As the number of components increased from 1 to 3, R^2^ rose substantially from 0.85418 to 0.99538, approaching nearly 1. This suggested that with more components, the model captures underlying patterns in the volatile compound data more completely, demonstrating a strong capacity to explain overall variability. Q^2^ reflected the model’s predictive performance for new samples (Saccenti and Timmerman, 2016). A value closer to 1 indicated better generalizability and accuracy in predicting unknown samples. When the number of components increased from 1 to 3, Q^2^ jumped from 0.60223 to 0.96514, approaching an ideal value. This indicated that the model exhibits excellent predictive ability for group differences in volatile compounds in new samples, rather than merely overfitting the training data. In conclusion, as the number of components increased, both the goodness-of-fit (R^2^) and predictive performance (Q^2^) of the PLS-DA model significantly approached ideal values, demonstrating robust fitting and generalization capabilities. The gradual improvement in classification accuracy further supports the model’s effectiveness in discriminating group differences based on volatile compounds.

### 3.5 Key aroma components and their aroma characteristics in fermented products of different bacterial species

The flavor characteristics of 12 fermented samples were analyzed based on Odor Activity Value (OAV) and Relative Odor Activity Value (ROAV) data in Supplementary Table S3. OAV is defined as the ratio of the concentration of a compound to its odor threshold; an OAV ≥ 1 suggests that the compound may contribute to the overall aroma (Zhu et al., 2016). ROAV is used to compare the relative contribution of different aroma components to the overall flavor profile, with the compound having the highest ROAV generally considered the key aroma substance (Fang et al., 2024). Seventy-four volatile aroma compounds were detected, primarily including alcohols, aldehydes, ketones, acids, and esters in Supplementary Table S3. Alcohols such as 1-Hexanol (grassy, almond-like aroma) and 1-Octen-3-ol (mushroom-like aroma) were widely present, with OAVs mostly ranging between 10 and 100, contributing fundamental green and fungal aroma. Aldehydes including (E)-2-Nonenal (fatty, green aroma), (E, E)-2,4-Nonadienal (fatty aroma), and Nonanal (citrus, soapy aroma) exhibited very high OAV values in multiple samples, indicating strong odor activity and significant flavor impact, and were identified as key components shaping the dominant aroma profiles. Among ketones, 2,3-Butanedione (sweetness aroma) was the most prominent. Acids and esters generally showed lower OAVs and contributed less to the overall flavor.

Each strain demonstrated a distinct aroma profile: PS1: Characterized by green and fatty aroma, dominated by (E)-2-Nonenal (OAV = 368.42, fatty, green), with significant contributions from 1-Hexanol (OAV = 26.79, green, almond) and 1-Octen-3-ol (OAV = 53.33, mushroom). The aroma was relatively simple and fresh. PS2: Exhibited intense fatty and citrus-soapy aroma, with extremely high levels of (E)-2-Nonenal (OAV = 1578.95) as the most dominant feature, supported by Nonanal (OAV = 200, citrus, soapy) and Hexanal (OAV = 42.86, green), forming a complex aroma profile. YL2: Balanced profile combining fatty, green, and fruity aroma. Although (E)-2-Nonenal (OAV = 1315.79) remained dominant, contributions from 1-Hexanol (OAV = 23.21, green) and 2-Octanol (OAV = 19.23, fruity, soapy) resulted in a softer and richer flavor compared to the PS and YS series. YS2: Highly intense and complex aroma marked by explosive creamy and fatty aroma. Both 2,3-Butanedione (OAV = 15593.22, creamy) and (E)-2-Nonenal (OAV = 1578.95, fatty) ranked among the highest across all samples, with 1-Octen-3-ol (OAV = 140, mushroom) providing a deep background note, making this sample the most potent in flavor intensity. YS3: Similar to YS2, displaying strong fatty and mushroom aromas. Core components included (E)-2-Nonenal (OAV = 1526.32) and 1-Octen-3-ol (OAV = 140), accompanied by 1-Hexanol (OAV = 67.86, green) and (E)-2-Octenal (OAV = 76.67, nutty, fatty), resulting in high complexity. ML1: Relatively mild profile featuring sour, green, and slight fatty aroma. (E)-2-Nonenal (OAV = 315.79) and 1-Hexanol (OAV = 14.29) provided the base aroma, while Acetic acid (OAV = 32.31, vinegar) contributed a distinct sourness. MS1: Dominated by green, soapy, and oily aroma. Hexanal (OAV = 7.14, green), Nonanal (OAV = 22.58, citrus, soapy), and 1-Octen-3-ol (OAV = 60, mushroom) were key contributors, with (E, E)-2,4-Nonadienal (OAV = 259.74, fried fat) adding an oily nuance. MS2: Exhibited a balanced profile of green and fatty aroma with a slight soapy impression. (E)-2-Nonenal (OAV = 684.21) and 1-Hexanol (OAV = 30.36) were primary contributors, while 2-Octanone (OAV = 2.19, soapy, fruity) provided a unique tone. YL1: Dominated by an intense creamy aroma, with 2,3-Butanedione (OAV = 10847.46, creamy) as the absolute core, supported by 1-Hexanol (OAV = 58.93, green) and 1-Octen-3-ol (OAV = 73.33, mushroom), forming a complex blend of creamy, green, and fungal aroma. YS1: Similar to YL1, dominated by a strong creamy note from 2,3-Butanedione (OAV = 15593.22), with support from (E)-2-Nonenal (OAV = 263.16, fatty, green) and 1-Hexanol (OAV = 25.00, green), resulting in a rich flavor. MS4: Predominantly fatty and green aroma, mainly contributed by (E)-2-Nonenal (OAV = 578.95), with significant support from (Z)-2-Decenal (OAV = 160, fatty, green). MS6: Exhibited an extremely intense creamy and citrus-soapy profile, being one of the most potent samples. 2,3-Butanedione (OAV = 39152.54, creamy) was present at remarkable levels, accompanied by high amounts of Nonanal (OAV = 422.58, citrus, soapy) and 1-Hexanol (OAV = 80.36, green), forming a rich and complex aroma.

Based on odor activity value (OAV) analysis and metabolic profiling, the aroma characteristics of the fermented samples can be clearly classified into two distinct types according to their dominant metabolic pathways. The ketone-dominant type, represented by strains such as YS1, YL1, and MS6, is characterized by extremely high OAVs of 2,3-butanedione (ranging from 10,800 to over 39,000), which contributes a strong, sweet, buttery aroma and constitutes the most dominant sensory attribute in this group. In contrast, the aldehyde-dominant type, including strains PS2, YS2, YL2, MS1, MS2, MS4, YS3, ML1, and PS1, exhibits significant accumulation of long-chain aldehydes, particularly (E)-2-nonenal (OAV 260–1,580), often accompanied by nonanal or (E, E)-2,4-nonadienal, collectively imparting a fresh, fatty, and citrus-soapy flavor profile. These fundamental differences reflect the divergence in the core metabolic pathways of the strains: the former is associated with a high-flux ketogenesis pathway, while the latter reflects active fatty acid oxidation. Additionally, alcohols such as 1-hexanol and 1-octen-3-ol provide supporting grassy and mushroom notes in both types, whereas acids and esters contribute minimally due to their lower OAVs. This classification not only clarifies the sensory structure but also points to inherent differences in the activity and expression regulation of key metabolic enzymes, which govern the proportion and quantity of critical aroma compounds, including aldehydes, ketones, and alcohols, thereby shaping the final flavor phenotype (Hao et al., 2025).

#### 3.5.1 Analysis of key volatile organic compound metabolic pathways of different bacterial species

The significant variations in aroma profiles observed among different fermentation samples are primarily attributed to differences in microbial metabolism. The synthesis of aroma compounds does not proceed independently but rather through an interconnected network centered around key metabolic nodes such as carbohydrates, fatty acids, proteins (Yu et al., 2025).

Based on KEGG enrichment and pathway analyses, the biosynthesis of key aroma components was shown to rely on the coordination of several core metabolic pathways, including pyruvate metabolism, glycolysis, fatty acid biosynthesis, butanoate metabolism, and the glyoxylate cycle in Figures 4E,F. Among these, pyruvate metabolism serves as a central hub for aroma formation. Pyruvate metabolism is converted by *α*-acetolactate synthase into α-acetolactate, which then undergoes spontaneous oxidative decarboxylation to 2,3-butanedione, a compound associated with a pronounced creamy aroma (Ogundipe et al., 2025; Syu, 2001). This pathway was found to be highly active in samples such as YS2 and MS6, accounting for their dominant ketone-like flavors (Supplementary Figure S1). Additionally, pyruvate can be transformed into Acetyl-CoA via the pyruvate dehydrogenase complex. Acetyl-CoA may then be hydrolyzed to acetate or serve as a precursor for fatty acid biosynthesis (Ogundipe et al., 2025). Fatty acid biosynthesis and subsequent oxidative degradation constitute another major route for the generation of key aroma compounds. Polyunsaturated fatty acids produced microbially are cleaved oxidatively by lipoxygenase or peroxidase, resulting in the formation of C6 and C9 aldehydes and alcohols. For example, linoleic acid can be transformed via the Lipoxygenase Pathway and hydroperoxide lyase into hexanal (imparting a green odor) and (E)-2-nonenal (imparting fatty and cucumber-like odors) (Liu et al., 2024; Ogundipe et al., 2025). This mechanism explained the notable accumulation of aldehydes in samples such as PS1, PS2, and MS4. Glyoxylate and dicarboxylate metabolism was identified to play an essential anaplerotic role under conditions where short-chain acids or ethanol act as carbon sources (Strijbis and Distel, 2010). The glyoxylate cycle enables microorganisms to utilize acetate as a carbon source for generating oxaloacetate, thereby replenishing TCA cycle intermediates and supporting biosynthetic reactions (Strijbis and Distel, 2010). This pathway is likely responsible for the high acetate accumulation observed in sample ML1 and may facilitate metabolic maintenance in low-carbon environments. Furthermore, intermediates of butanoate metabolism can act as precursors for the synthesis of esters and short-chain keto acids, indirectly shaping the aroma profile. In addition, the degradation of aminobenzoate, taurine and hypotaurine metabolism, pinene, camphor and geraniol degradation, sulfur metabolism and so on have exerted profound effects on the fermentation metabolism of the bacteria, which need further study. Future research may focus on metabolic engineering strategies for the rational optimization of flavor characteristics. These approaches provide a robust theoretical foundation and practical methodologies for the targeted development of high-quality fermented foods.

## 4 Conclusion

In this study, 12 aroma-producing microbial strains were isolated from tobacco leaves and root soil and identified as members of the genera Enterobacter, Burkholderia, and Pseudomonas using ITS and 16S rRNA gene sequencing. Fermentation profiling indicated that although some Enterobacter strains exhibited high amylase activity conducive to starch degradation, all isolates showed limited cellulase activity and ethanol tolerance.

The result of sensory evaluation and HS-SPME/GC–MS analysis revealed considerable divergence in flavor profiles among strains. A total of 174 volatile compounds were detected and categorized into nine chemical classes. Alcohols were the most abundant, followed by aldehydes, ketones, alkanes, esters, acids, alkenes, phenols, and others. Notably, 2,3-butanediol and ethanol were found in high concentrations, reaching 40.1 μg/g in MS4 and 26.02 μg/g in MS6, respectively. Odor Activity Value (OAV) analysis further identified 74 compounds as odor-active, among which ketones (2,3-butanedione) and aldehydes ((E)-2-nonenal, (E, E)-2,4-Decadienal) were the most influential, contributing creamy, fatty, and green aroma. Esters and acids played a secondary role in overall aroma perception. Based on volatile profiles and OAV data, the strains were classified into two functional aroma types: Ketone metabolic type (YS1, MS6, YL1), Aldehyde metabolic type (PS2, MS1, MS2, MS4, YS2, YL2, YS3, ML1, PS1). These variations were mainly attributed to differences in central carbon metabolic flux distribution, particularly through the pyruvate metabolism and lipoxygenase pathways. The findings confirmed that targeted flavor modulation in fermentation processes can be achieved through selection of strains with defined metabolic traits, offering a scientific basis for microbial flavor design. Certainly, this study had certain limitations in terms of the consideration of volatile matrices and the accuracy of quantification, which require further refinement and optimization in future research.

The future work will focus on metabolic engineering of key enzymes to enhance flavor yield, explore multi-strain synergistic fermentation for complex aroma blending, and validate the industrial applicability of superior strains in real fermentation systems such as tobacco. Future enhancement strategies will focus on metabolic engineering of key aroma biosynthesis genes, co-cultivation with specialized lignocellulolytic microbes, and process optimizations such as fed-batch fermentation to overcome existing constraints in enzymatic activity and ethanol tolerance. These improvements are designed to target specific aroma-relevant metabolic nodes-including the Ehrlich pathway for generating amino acid-derived alcohols, esterification routes that enhance fruity notes, and *β*-glucosidase-catalyzed liberation of glycosidically-bound terpenoids. These directions will advance both theoretical and practical applications of microbial aroma formation in engineering. In addition, the biosafety of the screened microbial strains is a fundamental prerequisite for their potential translation into industrial applications. To ensure biosafety, candidate strains will undergo comprehensive safety evaluation prior to scale-up, encompassing genomic screening, precise taxonomic identification, and phenotypic assessment, coupled with a process control strategy that prioritizes either the use of inactivated biomass or enzyme preparations, or implementation of closed-system fermentation followed by terminal sterilization. Any future industrial implementation will furthermore adhere to all relevant national and international biosafety and food safety regulations.

## Data Availability

The original contributions presented in the study are publicly available. This data can be found at: National Center for Biotechnology Information (NCBI) under the accession numbers SRR37231021(MS4), SRR37231020(MS6), SRR37231017(ML1), SRR37231016(MS1), SRR37231015(MS2), SRR37231014(PS1), SRR37231013(PS2), SRR37231012(YL1), SRR37231011(YS1), SRR37231010(YL2), SRR37231019(YS2), and SRR37231011(YS3). The 16S rRNA sequencing data have been deposited in the NCBI SRA database under BioProject accession number PRJNA1423604.
