# Supplementary material for: Exploring the potential of tobacco microorganisms for enhancing the flavor of naturally fermented essential oils
Source: Front Microbiol. 2026 Oct 1;17:1780070. doi: 10.3389/fmicb.2026.1780070 (PMC13668867; doi:10.3389/fmicb.2026.1780070)
Supplement: Supplementary file 1 [file Supplementary_file_1.DOCX]

**Figure S1** Enrichment analysis of characteristic metabolic pathways in all aroma-producing bacteria.

**PS1**

**PS2**

**YL2**

**YS2**

**YS3**

**MS4**

**MS6**

**ML1**

**MS1**

**MS2**

**YL1**

**YL2**
