# Supplementary material for: Exploring the potential of tobacco microorganisms for enhancing the flavor of naturally fermented essential oils
Source: Front Microbiol. 2026 Oct 1;17:1780070. doi: 10.3389/fmicb.2026.1780070 (PMC13668867; doi:10.3389/fmicb.2026.1780070)
Supplement: Supplementary file 2 [file Table_1.DOCX]

**Table S1** Primers

| Sequencing regions | Name of the primer | Primers | Identify |
| --- | --- | --- | --- |
| ITS1-ITS4 | ITS1 | TCCGTAGGTGAACCTGCGG | fungus |
|  | ITS4 | TCCTCCGCTTATTGATATGC |  |
| 27F-1492R | 27F | AGAGTTTGATCCTGGCTCAG | bacterium |
|  | 1492R | GGTTACCTTGTTACGACTT |  |
